# Dietary uridine improves lipid homeostasis in high-fat diet-induced obese mice by regulating liver gene expression and metabolomic profiles

**DOI:** 10.3389/fnut.2025.1651993

**Published:** 2025-09-22

**Authors:** Yilin Liu, Huihui Zhang, Xudong Yang, Chunyan Xie

**Affiliations:** ^1^Henan Key Laboratory of Zhang Zhongjing Formulae and Herbs for Immunoregulation, Zhang Zhongjing College of Chinese Medicine, Nanyang Institute of Technology, Nanyang, China; ^2^Tianjin Key Laboratory of Animal Molecular Breeding and Biotechnology, Tianjin Livestock and Poultry Health Breeding Technology Engineering Canter, Institute of Animal Science and Veterinary, Tianjin Academy of Agricultural Sciences, Tianjin, China; ^3^Tianjin Key Laboratory of Agricultural Animal Breeding and Healthy Husbandry, College of Animal Science and Veterinary Medicine, Tianjin Agricultural University, Tianjin, China

**Keywords:** uridine, lipid metabolism, apoptosis, metabolomics, obese mice

## Abstract

**Introduction:**

Obesity is caused by excessive storage of adipose tissue and leads to metabolic disorders. Uridine exerts modulatory effects on lipid metabolism, but the regulatory mechanism in obesity needs further research.

**Methods:**

In this study, the effects of uridine supplementation on lipid metabolism were investigated in high-fat diet-induced obese mice. Mice aged at 8 weeks were randomly grouped to receive a control diet (CON, *n* = 10) or a high-fat diet (HF, *n* = 24). After 6 weeks of feeding, the HF group was further divided, with half receiving 0.4 mg/mL uridine supplementation in drinking water (HUR, *n* = 12) for an additional 4 weeks, while the remaining HF mice continued without intervention.

**Results:**

The results showed that the uridine supplement reduced the liver weight and intra-abdominal white adipose tissue weight in obese mice (*p <* 0.05). Treatment with uridine and free fatty acid resulted in a significant increase in late and total apoptosis, accompanied by a decrease in early apoptosis of mouse liver organoids (*p* < 0.05). Moreover, uridine lowered serum levels of triglycerides (TG), total cholesterol (TC), high-density lipoprotein (HDL), leptin, and liver TG content (*p <* 0.05). In obese mice fed with uridine, the expression of key genes involved in lipid transport [activated fatty acid translocase/cd36 (*Fat/cd36*) and low-density lipid receptor (*Ldlr*)], pyrimidine *de novo* synthesis [dihydroorotate dehydrogenase (*Dhodh*)], pyrimidine metabolism [uridine phosphorylase 2 (*Upp2*), ribonucleoside-diphosphate reductase subunit M2 (*Rrm2*), and thymidine kinase 1 (*Tk1*)] was improved (*p <* 0.05). Furthermore, liver metabolomic analysis identified 37 differential metabolites between the HF and HUR groups, primarily enriched in arachidonic acid metabolism and *α*-linolenic acid metabolism.

**Discussion:**

These findings indicated that uridine supplementation can improve lipid metabolism in obese mice by regulating hepatic gene expression and metabolic pathways.

## 1 Introduction

Obesity is defined as abnormal and excessive fat accumulation induced by nutritional imbalance (1). It has become a significant public health issue worldwide over the last four decades. The World Health Organization (WHO) reported that as of 2022, the global number of obese people had exceeded 1 billion (2). Obesity may develop and increase the risk of dyslipidemia, non-alcoholic fatty liver disease, insulin resistance, cardiovascular disease, and chronic inflammation (3). Hepatic steatosis can further progress to non-alcoholic steatohepatitis and eventually lead to end-stage liver diseases such as cirrhosis and liver cancer (4). A high-fat (HF) diet inhibited hepatic energy expenditure, induced steatosis and oxidative stress, and was also a widely used model of obesity (5). Therefore, it is essential to treat obesity and maintain liver function. The use of natural sources has emerged as a promising strategy for the prevention and management of obesity, such as natural polysaccharides, terpenoids, and flavonoids (6, 7).

Uridine, a pyrimidine nucleoside, is abundant in milk, such as bovine milk (14.67–132.6 μmol/L), caprine milk (17.9–78.5 μmol/L), ovine milk (67.8–115.3 μmol/L), and human milk (0.5–26 μmol/L) (8). Notably, physiological regulation established circulating uridine as a signal of the nutritional status of an organism and suggested a role for uridine in the control of energy balance (9). Uridine regulates liver energy homeostasis through promoting the biosynthesis of membrane phospholipid, enhancing fatty acid *β*-oxidation and lipid glycosylation, modulating the protein acetylation profile, and affecting protein glycosylation (10–12). Recently, it has been reported that plasma uridine governs energy homeostasis and thermoregulation in a mechanism involving adipocyte-dependent uridine biosynthesis and leptin signal (13). Furthermore, activation of uridine production in adipocytes can enhance lipolysis and invoke a potential anti-obesity strategy through the induction of a futile biosynthetic cycle (14).

Previous studies have shown that uridine regulates liver fatty acid composition and lipid metabolism in piglets and mouse models (15, 16). However, limited data are available on the role of uridine in regulating lipid metabolism in the context of obesity. It was hypothesized that uridine administration could alleviate obesity-related lipid metabolic disorders in obese mice, resulting in reduced body fat content. Therefore, in the current study, an HF diet-induced obesity model was used to elucidate the effect of dietary uridine on lipid homeostasis.

## 2 Methods and materials

### 2.1 Experimental animals and diets

Male C57BL/6 J mice were purchased from SLAC Laboratory Animal Central (Changsha, China). As suggested by the animal welfare protocol, all efforts were made to minimize animal suffering and to use only the number of animals necessary to produce reliable scientific data. All animals were housed in a climate-controlled room (temperature, 25 ± 2 °C; relative humidity, 45–60%; lighting cycle, 12 h light/dark cycle with light provided 08:00–20:00) and had free access to food and drinking water during the duration of the experiments.

**Figure 1:**
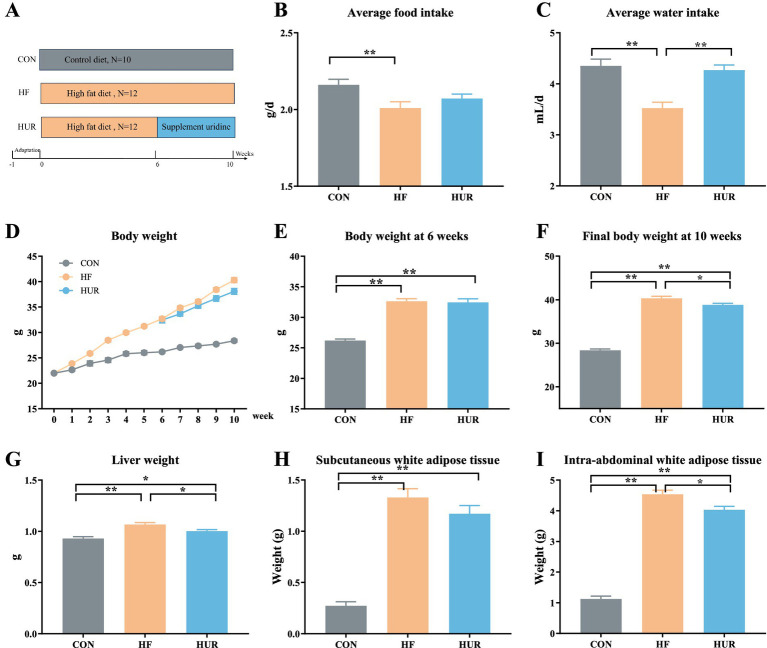
Effects of uridine on **(A)** schema showing the animal groups and treatments, **(B)** average food intake, **(C)** average water intake, **(D)** body weight, **(E)** body weight at 6 weeks, **(F)** final body weight, **(G)** liver weight, **(H)** subcutaneous white adipose tissue weight, and **(I)** intra-abdominal white adipose tissue weight (*n* = 10–12). CON, control diet group; HF, high-fat diet group; HUR, high-fat diet + 0.4 mg/mL uridine in drinking water for the last 4 weeks. *p < 0.05, **p < 0.01.

After 1 week of adaptation, C57B/6 J male mice aged at 8 weeks were randomly divided into the CON group (received control diet, *n* = 10), the HF group (received HF diet, *n* = 12), and the HUR group (received high-fat diet and supplemented with 0.4 mg/mL uridine in drinking water for the last 4 weeks, *n* = 12) for 10 weeks (17). The schematic diagram of the animal group and treatment is shown in Figure 1A. The consumption of feed and drinking water was measured and recorded daily to calculate the average feed intake and water intake. The drinking water was renewed every 2 days. An average of 4.27 ± 0.57 mL/d of uridine water was drunk per mouse in the HUR group (Figure 1C). The control diet consisted of 10% (kcal%) fat, 20% (kcal%) protein, and 70% (kcal%) carbohydrate (D12450B; Research Diets, Inc., New Brunswick, NJ, USA); the HF diet consisted of 60% (kcal%) fat, 20% (kcal%) protein, and 20% (kcal%) carbohydrate (D12492; Research Diets, Inc.). Uridine (purity ≥ 99.90%) was provided by Meiya Pharmaceutical Co., Ltd. (Hangzhou, China). At the end of the experiment, the mice were sacrificed after being deeply anesthetized. Blood was collected from the mice by retro-orbital bleeding, centrifuged for 10 min at 16,000 × *g*, frozen on dry ice, and stored at −80 °C. The liver, subcutaneous, and intra-abdominal white adipose tissues (including the epididymal, perirenal, and mesenteric white adipose tissues) were weighed. The liver was snap-frozen in liquid nitrogen and then stored at −80 °C for further analysis.

### 2.2 Mouse liver organoid culture

Mouse duct cells were isolated from mouse liver using a digestion solution composed of DNase-1 (100 μg/mL; Sigma-Aldrich, St. Louis, MO, USA), collagenase type XI (125 μg/mL; Sigma-Aldrich, St. Louis, MO, USA), dispase (125 μg/mL; Thermo Fisher Scientific, Shanghai, China), and 1% FBS in DMEM. Following isolation, bile ducts were embedded in Matrigel (BD Bioscience, San Diego, CA, USA) and seeded in a 24-well plate. Cultures were maintained in expansion medium, refreshed every 2–3 days, and passaged weekly at a 1:2–1:4 ratio. The expansion medium consisted of the medium previously reported (17).

### 2.3 Free fatty acid preparation

Oleic acid (OA) and palmitic acid (PA) (Sigma-Aldrich, St. Louis, MO, USA) were complexed with bovine serum albumin (BSA) (Sigma-Aldrich, St. Louis, MO, USA) and sterile-filtered. The free fatty acid (FFA) mixture was dissolved in the medium at a final concentration of 600 μM (OA: PA = 2:1) to induce lipid accumulation according to our previous research (17).

### 2.4 Cell apoptosis assay

Mouse liver organoids collected from 12-well plates were digested into single cells using trypsin, followed by suspension in complete culture medium and centrifugation. The Annexin V-FITC/PI apoptosis detection kit was used to detect the apoptosis of uridine and free fatty acid preparation-treated mouse liver organoids according to the manufacturer’s instructions (BD Biosciences, San Diego, CA, USA). Briefly, cells were resuspended in 195 μL of Annexin V-FITC binding buffer. Then, 5 μL of Annexin V-FITC reagent was added and gently mixed, followed by incubation on ice for 10 minutes in the dark. Subsequently, 5 μL of propidium iodide (PI) staining solution was added, and the cells were further incubated for 15 minutes at room temperature in the dark. Finally, 200 μL of binding buffer was added prior to flow cytometry analysis. The ratio of apoptotic cells was determined using a FACS instrument (BD FACSCanto II, San Diego, CA, USA). Cells exhibiting PI-negative and Annexin V-FITC-positive staining were considered to be in the early stages of apoptosis, whereas cells with positivity for both PI and Annexin V-FITC were identified as late apoptotic.

### 2.5 Biochemical analyses

The concentrations of serum triglycerides (TG), total cholesterol (TC), and high-density lipoprotein (HDL) were determined by an Automated Biochemistry Analyzer (Synchron CX Pro, Beckman Coulter, Fullerton, CA, USA) according to the commercial kits and manufacturer’s instructions (Beijing Chemclin Biotech Co., Ltd., Beijing, China). The serum leptin was assayed using mouse leptin ELISA kits (CUSABIO Co., Ltd., Wuhan, China) according to the manufacturer’s instructions.

Liver tissue samples were isolated after mice were killed by cervical dislocation, rinsed with 0.9% NaCl, snap-frozen in liquid nitrogen, and stored at −80 °C until further analysis. The concentrations of liver TG and total protein were determined by a multimode reader (Tecan Trading AG, Switzerland) based on the commercial assay kits provided by Applygen Technologies Inc. (Beijing, China) for the tissue TG content assay kit and the trace protein quantification kit (BCA method).

### 2.6 RNA isolation and quantification of gene expression

Total RNA was isolated from the liver tissue using Trizol Reagent (Invitrogen, Carlsbad, CA, USA) according to the manufacturer’s instructions. RNA concentrations and A260/A280 ratios were determined on a NanoDrop ND-1000 spectrophotometer (Thermo Fisher Scientific, Wilmington, DE, USA). The total RNA was reverse-transcribed into cDNA using the PrimeScript™ RT reagent kit (Takara Biomedical Technology, Japan). Quantitative real-time RT-PCR (qRT-PCR) was performed with SYBR Green I Dye (Thermo Fisher Scientific, New York, USA) using the LightCycler 480II real-time PCR system (Roche, Basel, Switzerland). The PCR cycling conditions were as follows: 95 °C for 5 min and 98 °C for 2 min, followed by 40 cycles of 5 s at 98 °C, 5 s at 60 °C, 10 s at 95 °C, and a final step of 1 s at 65 °C. The primers for the genes are listed in Table 1. The gene expression results were expressed as the mean relative mRNA level (18).

**Table 1:** Primers used for quantitative reverse transcription PCR.

Gene	Forward primer 5’-3’	Reverse primer 5’-3’	Accession number	Product length
*Fat/cd36*	ACGCAGCCTCCTTTCCACCTTT	CGAACACAGCGTAGATAGACCTGCA	NM_001159558.1	90
*Ldlr*	CCACAGAACTGCCAGGGCCG	GAATTCATCAGGTCGGCAGGT	NM_001252659.1	186
*Vldlr*	GAGCCCCTGAAGGAATGCC	CCTATAACTAGGTCTTTGCAGATATGG	XM_021212964.2	83
*Cad*	AGAAAGGGACAGAGCCGTCAG	ATCCAGAGCACAGATCCGAGG	XM_006504092.3	128
*Dhodh*	CGTTCGGCTGTCCAATCAAC	GTAGAAATGGTCGTCCCCCG	NM_020046.3	233
*Umps*	GGCGACAGTTATCTGCTCAGC	CGTCCTCAATGACCAGACAGG	NM_009471.3	131
*Upp2*	CGGTTGGAGGGAGATGGAGAA	AATGGAAATGGAGGGGATGCC	XM_006498411.3	123
*Rrm2*	CTGTTTCTATGGCTTCCAAAT	TTCTTCTTCACACAAGGCATT	NM_009104.2	141
*Cmpk2*	CTGCTTAACTCTGCGGTGTTC	CTTTCTGGACCTCCTTTGGGC	NM_020557.4	130
*Tk1*	CGGAGAGTGTGGTGAAGCTCA	CACGGAGTGATACTTGTCGGC	XM_006533150.3	125
*Tk2*	TCCAAGACCCCATCACTCTCTC	TGACTTCTTCATGCTCGTGGTC	XM_006533150.3	125
*Gapdh*	TTGTGATGGGTGTGAACCACGA	TCTTCTGGGTGGCAGTGATGG	NM_008084.2	168

Fat/cd36, fatty acid translocase; Ldlr, low-density lipid receptor; Vldlr, very low-density lipoprotein receptor; Cad, carbamoyl-phosphate synthetase 2, aspartate transcarbamoylase, and dihydroorotase; Dhodh, dihydroorotate dehydrogenase; Umps, uridine monophosphate synthetase, Upp2, uridine phosphorylase 2, Rrm2, ribonucleoside-diphosphate reductase subunit M2; Cmpk2, cytidine/uridine monophosphate kinase 2; Tk1, thymidine kinase 1, and Tk2, thymidine kinase 2.

### 2.7 Liver untargeted metabolomic analyses

Samples that contained 100 mg of liver tissue powder were cryo-weighed, and 80% methanol (v/v H2O) was added at a ratio of 300 μL dissolver/100 mg tissue. The samples were shaken for 20 min at room temperature and centrifuged at 14,000 × *g* for 15 min at 4 °C. Supernatants were filtered (0.2 μm) and stored at −20 °C until the next analysis. LC–MS/MS analyses were performed using a Thermo Vanquish UHPLC with an Accucore Vanquish C18 column (50 × 2.0 mm, 1.5 μm) (19). The mobile phases of solutions A and B were composed of 95% acetonitrile with 0.1% formic acid and 10 mM ammonium acetate and 50% acetonitrile with 0.1% formic acid and 10 mM ammonium acetate in the positive mode. The flow rate was at 300 μL/min, which consisted of 2% B for 1 min, 50% B for 16 min, 50% B for 0.5 min, 2% B for 0.5 min, and 2% B for 2 min.

For the mass spectrometric assay, a mass spectrometer detector QE HF-X (Thermo Fisher Scientific Inc., Massachusetts, CA, USA) was used to analyze the metabolite ions. Electrospray ionization (ESI) settings are as follows: spray voltage: 3.2 kV; sheath gas flow rate: 35 arb; aux gas flow rate: 10 arb; and capillary temperature: 320 °C. Estimation was performed in positive and negative modes with a scan time of 20 min. MS/MS secondary scan is a data-dependent scan. Spectral data from all peaks were accumulated in the range of 100–1,500 *m/z*. Molecular formulas were determined by using Xcalibur™ software (Thermo Fisher Scientific, New York, USA), and the identification of constituent substances was performed by Compound Discoverer software, which was confirmed by searching online libraries of mzVault, ChemSpider, and mzCloud.

Processed datasets were analyzed by multivariate statistical analysis using SIMCA+13.0 (Umetrics, Umea, Sweden). Partial least squares discriminant analysis (PLS-DA) was used to visualize discrimination among samples. The quality of PLS-DA models was evaluated by R^2^Y and Q^2^; the goodness of fit measure was quantified by R^2^Y; and the predictive ability was indicated by Q^2^. Validation and reliability of PLS-DA models were rigorously confirmed by a permutation test (*n* = 200). For identification of metabolites contributing to the discrimination, the intensity differences of metabolites with a variable importance in the projection value (VIP) > 1.0, showing high relevance for explaining the differences among sample groups, were analyzed using t-tests (*p* < 0.05). Metabolic pathway analysis was employed using the MetaboAnalyst, and metabolites that showed significant change were mapped to the Kyoto Encyclopedia of Genes and Genomes (KEGG) pathways.

### 2.8 Statistical analysis

The data were expressed as mean ± standard error of mean (SEM), and the results were analyzed using one-way ANOVA, followed by the LSD test using SPSS 22.0. Differences were considered statistically significant at *p <* 0.05.

## 3 Results and discussion

### 3.1 Effects of uridine on body weight, food, and water intake in obese mice

During the experiment, the average food and water intake of the HF group was significantly lower than that of the CON group (*p <* 0.05; Figures 1B, C). However, the water intake was significantly increased in the HUR group compared with the HF group (*p <* 0.05). The body weight of mice during 10 weeks is shown in Figure 1D, and the initial body weight of mice was approximately 22 g. The body weights of mice both in the HF group (32.7 g) and the HUR group (32.5 g) exhibited a significant increase of over 20% compared to the CON group (26.2 g) at the 6th week (*p* < 0.01; Figure 1E), confirming the successful induction of obesity. Compared with the HF group (40.3 g), the final body weight at the 10th week of mice in the HUR group (38.8 g) showed a significant decrease (*p* < 0.05; Figure 1F). Obesity induces excessive storage of adipose tissue and leads to excessive accumulation of fat in other tissues, thereby posing a risk of developing metabolic disorders (20). A previous study showed that postprandial uridine metabolism was altered by obesity (21). The supplementation of uridine during both day and night significantly reduced body weight gain in the HF diet-fed mice (15). In the present study, supplementing with uridine through drinking water throughout the day also showed a decrease in body weight in HF diet mice. It may be suggested that the effect of uridine on the body weight of HF diet mice was not affected by the time of administration.

### 3.2 Effects of uridine on liver and adipose tissue weights in obese mice

Obesity induced by HF diet feeding in mice is typically characterized by dysregulated lipid metabolism and impaired liver function (22). The weight of the liver, subcutaneous white adipose tissues, and intra-abdominal white adipose tissues of mice in the HF group was higher than that in the CON group (*p <* 0.01; Figures 1G–I). As the primary site for carbohydrate and lipid biosynthesis, the liver plays a central role in regulating systemic glucose and lipid flux (23, 24). Notably, the HUR group presented a lower liver weight and intra-abdominal adipose tissue weight than the HF group (*p <* 0.05), although these parameters remained significantly different from those in the CON group (*p <* 0.05). These results indicated that uridine supplementation can ameliorate adipose tissue accumulation induced by HF feeding, but it is not sufficient to fully restore it to the same level as the CON group.

### 3.3 Effects of uridine on cell apoptosis in mouse liver organoids

**Figure 2:**
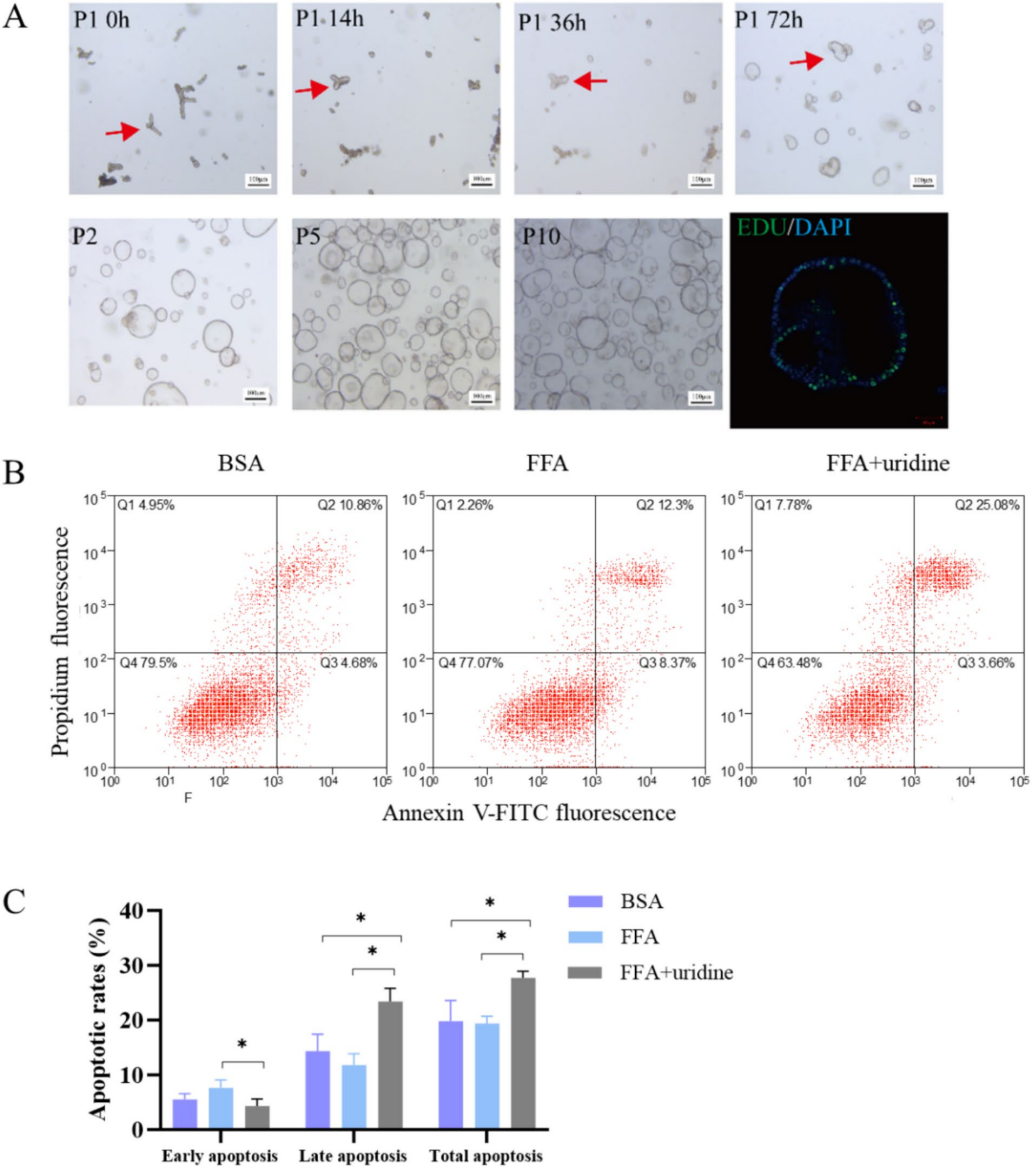
Mouse duct fragments formed spheres after 72 h, which were stably cultured in expansion medium from passage 1 (P1) to passage 10 (P10), with EDU staining confirming their proliferative activity **(A)**; The apoptotic results of liver organoids co-treated with fatty acids (24 h) and then uridine (48 h) were observed by flow cytometry after Annexin V-FITC/PI staining (*n* = 3) **(B)**; Quantitative analysis of apoptotic cell populations in each group **(C)**; BSA, bovine serum albumin; FFA, free fatty acid; *p < 0.05.

Mouse liver organoids were successfully cultured from mouse liver samples (Figure 2A). Following enzymatic digestion, bile duct fragments were observed in the supernatant. After 14 h of culture, biliary epithelial cells began to reorganize, forming spherical structures. By 72 h, complete reorganization resulted in the emergence of organoids displaying characteristic spherical morphology within Matrigel. The liver organoids could be stably cultured in expansion medium from passage 1 to passage 10, maintaining similar morphology with occasional folding and budding. After passaging, organoids demonstrated rapid proliferation and showed strong proliferative activity by 5-Ethynyl-2´-deoxyuridine (EDU) staining. The mouse liver organoids were seeded into 12-well plates with a density of 1 × 10^5^ cells/well for 24 h and incubated with FFA for 24 h and then with uridine for 48 h. The results of apoptosis analysis using flow cytometry showed that FFA treatment did not affect the apoptosis rate of liver organoid cells (Figures 2B,C). However, the addition of uridine alongside FFA treatment significantly increased the proportions of total apoptosis and late apoptosis while decreasing the proportion of early apoptosis (*p* < 0.05). Liver homeostasis is achieved by a tightly regulated steady state in cell turnover involving proliferation and apoptosis of hepatocytes. Increased numbers of apoptotic hepatocytes and apoptosis-associated degradation products were detected in NASH patients (25). Hepatocytes and bile duct cells are the first hepatic cell types to enter the cell cycle and proceed to mitosis. Proliferation subsides by days 5–7, while during the peak proliferation period, triglycerides accumulate in hepatocytes for 2–3 days (26). A previous study reported that 1 mM uridine significantly suppressed lipid accumulation induced by FFA exposure in the liver organoids (17). Similarly, uridine promotes the renewal of intestinal cells, and 50 mM uridine significantly promotes apoptosis of IPEC-J2 cells (27). The 1 mM and 10 mM uridine also inhibit the germination of intestinal organoids and suppress the stemness of intestinal stem cells, and this effect may be mediated through the mTOR pathway (28). The uridine exhibited higher distribution of G1/M phase of the cell cycle at 400 M compared with 0 M and reduced S-phases of the cell cycle compared with 0 and 100 M (*p* < 0.05) (29). It indicated that uridine is involved in the regulation of stem cell stemness and cell proliferation, regulating cell renewal.

### 3.4 Effects of uridine on serum lipids and leptin in obese mice

**Figure 3:**
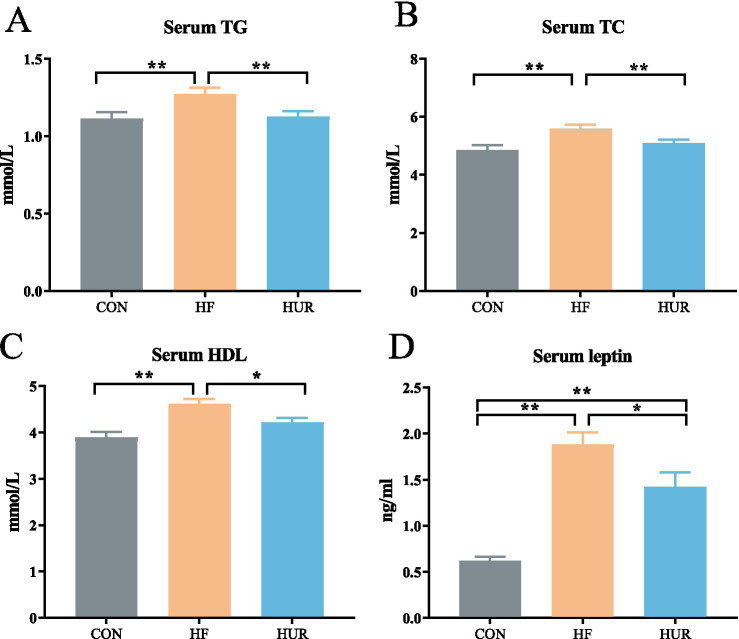
Effects of uridine on **(A)** serum TG, **(B)** serum TC, **(C)** serum HDL, and **(D)** serum leptin content (*n* = 10–12). TG, triglyceride; TC, total cholesterol; HDL, high-density lipoprotein; CON, control diet group; HF, high-fat diet group; HUR, high-fat diet + 0.4 mg/mL uridine in drinking water for the last 4 weeks. *p < 0.05, **p < 0.01.

Typical dyslipidemia of obesity includes elevated serum TG and TC (30, 31). In addition, the obesity level was consistent with the change of serum leptin, which was significantly increased after 12 weeks of HF feeding (32). As shown in Figure 3, consumption of an HF diet led to higher serum TG, TC, HDL, and leptin content than that in the CON group (*p <* 0.05). However, this increase was significantly decreased by the uridine supplement in obese mice (*p <* 0.05). Fasting-induced increases of plasma uridine occurred concurrently with high levels of lipolysis (13). Activation of uridine production in adipocytes can promote lipolysis, which suggests that uridine helps to alleviate obesity-related lipid metabolic disorder (14). Consistently, it has been reported that through the elevation of circulating uridine, *Lactobacillus* MR1 alleviates high-carbohydrate diet-induced hepatic lipid accumulation and oxidative stress (33). Similarly, a uridine supplement decreased hepatic lipid, serum glucose, triglyceride, and cholesterol in Nile tilapia with a high-carbohydrate diet (34).

### 3.5 Effects of uridine on TG content and lipid transporters in the liver of obese mice

**Figure 4:**
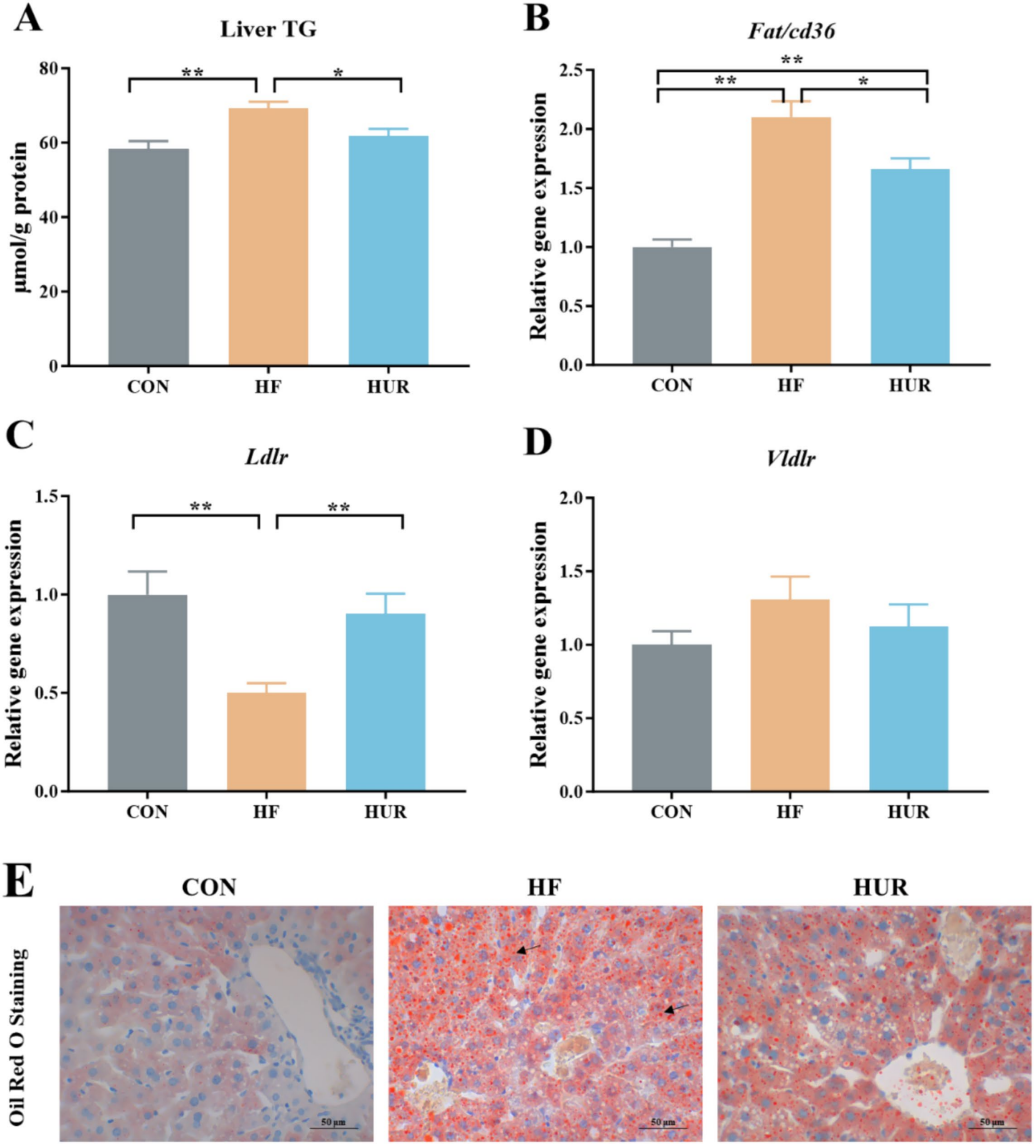
Effects of uridine on **(A)** liver TG content, **(B)** *Fat/cd36* gene expression, **(C)** *Ldlr* gene expression, **(D)** *Vldlr* gene expression, and **(E)** Oil red staining of the liver (*n* = 10–12). Fat/cd36, fatty acid translocase; Ldlr, low-density lipid receptor; Vldlr, very low-density lipoprotein receptor; CON, control diet group; HF, high-fat diet group; HUR, high-fat diet + 0.4 mg/mL uridine in drinking water for the last 4 weeks. *p < 0.05, **p < 0.01.

As shown in Figure 4, the HF diet increased the hepatic TG content of mice, and activated fatty acid translocase /cd36 (*Fat/cd36*) gene expression but inhibited low-density lipid receptor (*Ldlr*) mRNA levels when compared with those in the CON group (*p <* 0.05). Conversely, uridine supplementation significantly decreased the liver TG content and *Fat/cd36* expression and increased *Ldlr* expression in the liver of obese mice from the HUF group (*p <* 0.05). Oil red staining also showed that mice in the HF group presented greater lipid droplets in the liver when compared with those in the CON group, and uridine supplementation weakened the hepatic lipid droplet production in the HF group (Figure 4E). Importantly, uridine alleviated this typical dyslipidemia of obese mice after 4 weeks of administration, which was consistent with previous reports that uridine supplementation affected liver lipid accumulation (10, 35–37). The liver is involved in the uptake and secretion of fatty acids and TG (38). *Fat/cd36* is an important regulator of tissue free fatty acid (FFA) uptake from plasma and increases in the livers of obese subjects (23). Hepatocyte-specific deletion of *Cd36* protects against liver lipid accumulation induced by the HF diet in mice (39). In addition, *Ldlr* mRNA expression levels were decreased significantly following HF in the liver (40). Therefore, uridine supplementation may restore liver TG by regulating the expression of *Fat/cd36* and *Ldlr* genes in obese mice fed with an HF diet.

### 3.6 Effects of uridine on the relative gene expression related to pyrimidine *de novo* synthesis and metabolism in obese mice

**Figure 5:**
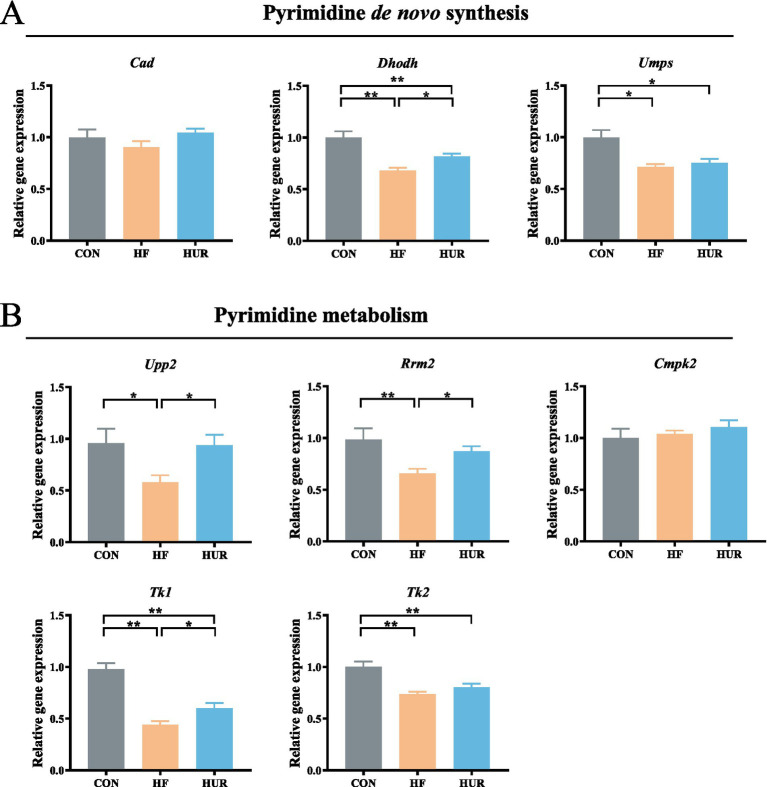
Effects of uridine on the relative expression of **(A)** pyrimidine *de novo* synthesis and **(B)** pyrimidine metabolism genes in the liver (*n* = 10–12). *Cad*, carbamoyl-phosphate synthetase 2, aspartate transcarbamoylase, and dihydroorotase; *Dhodh*, dihydroorotate dehydrogenase; *UMPS*, uridine monophosphate synthetase, *Upp2*, uridine phosphorylase 2, *Rrm2*, ribonucleoside-diphosphate reductase subunit M2; *Cmpk2*, cytidine/uridine monophosphate kinase 2; *Tk1*, thymidine kinase 1, and *Tk2*, thymidine kinase 2; CON, control diet group; HF, high-fat diet group; HUR, high-fat diet + 0.4 mg/mL uridine in drinking water for the last 4 weeks. *p < 0.05, **p < 0.01.

The relative gene expression related to pyrimidine metabolism in the liver is noted (Figure 5). Consumption of an HF diet significantly decreased dihydroorotate dehydrogenase (*Dhodh*), uridine monophosphate synthetase (*UMPS*), uridine phosphorylase 2 (*Upp2*), ribonucleoside-diphosphate reductase subunit M2 (*Rrm2*), and thymidine kinase 1 (*Tk1*) gene expression levels when compared with those in the CON group (*p <* 0.05). Especially, the expression of *Dhodh*, *Upp2, Rrm2*, and *Tk1* was higher in the HUR group than in the HF group (*p <* 0.05). Particularly, pyrimidine metabolism was abnormal in obese mice, such as low expression of UDP synthesis and conversion-related genes in the hypothalamus (41). Similarly, plasma uridine was significantly elevated after fasting for 4–24 h in healthy mice, but not in HF diet mice (13). Consistent with pyrimidine metabolism abnormalities observed in obese mice, our study revealed decreased expression of hepatic genes involved in pyrimidine metabolism and *de novo* synthesis, which was ameliorated by uridine supplementation, further supporting the regulatory role of uridine in metabolic disturbances induced by HFD.

### 3.7 Effects of uridine on liver metabolomics in obese mice

**Figure 6:**
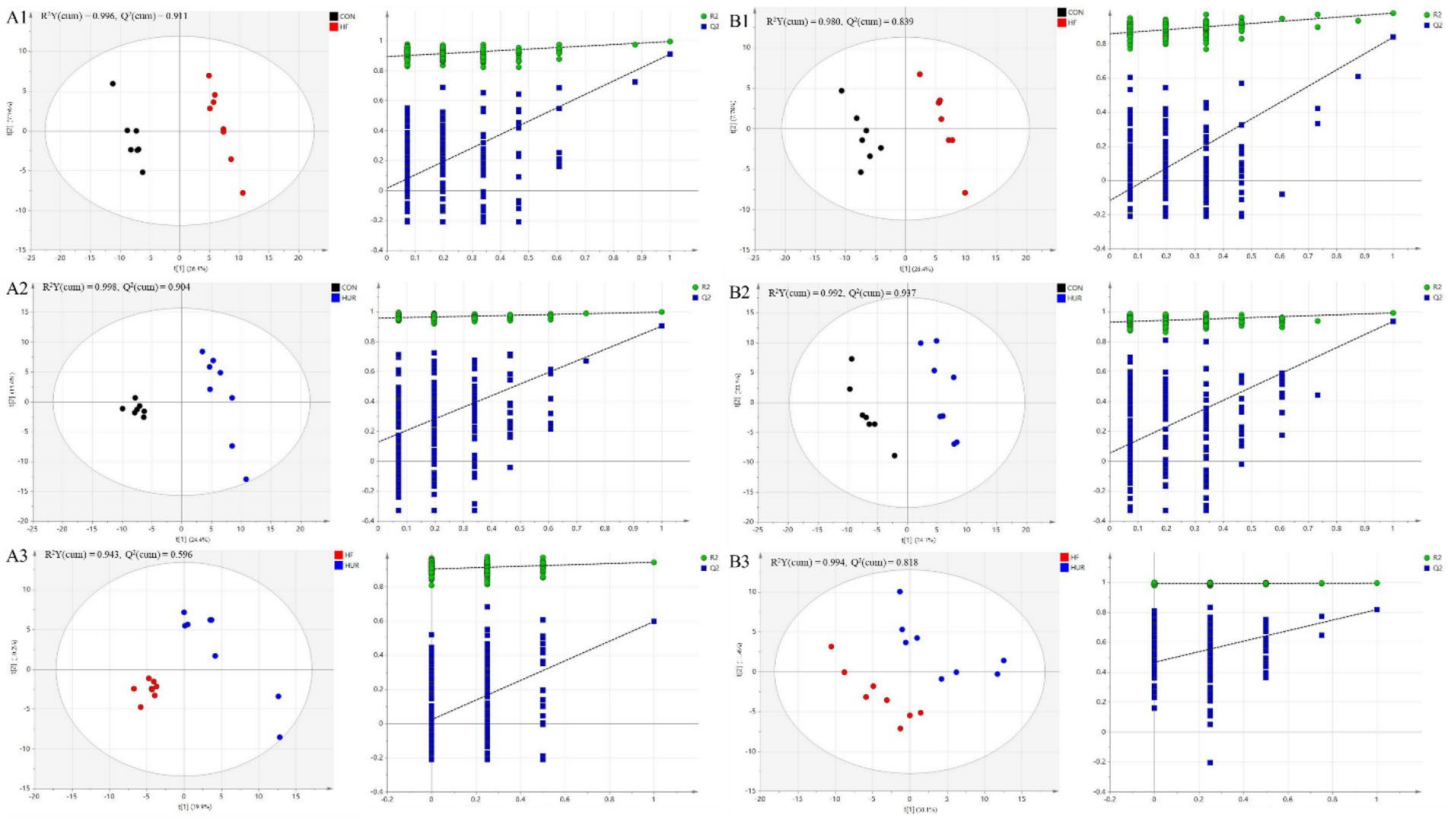
The score plots of partial least squares-discriminant analysis (PLS-DA) models and permutation test of the OPLS-DA models between groups in **(A1–A3)** positive mode and **(B1–B3)** negative mode (*n* = 7–8). CON, control diet group; HF, high-fat diet group; HUR, high-fat diet + 0.4 mg/mL uridine in drinking water for the last 4 weeks.

**Figure 7:**
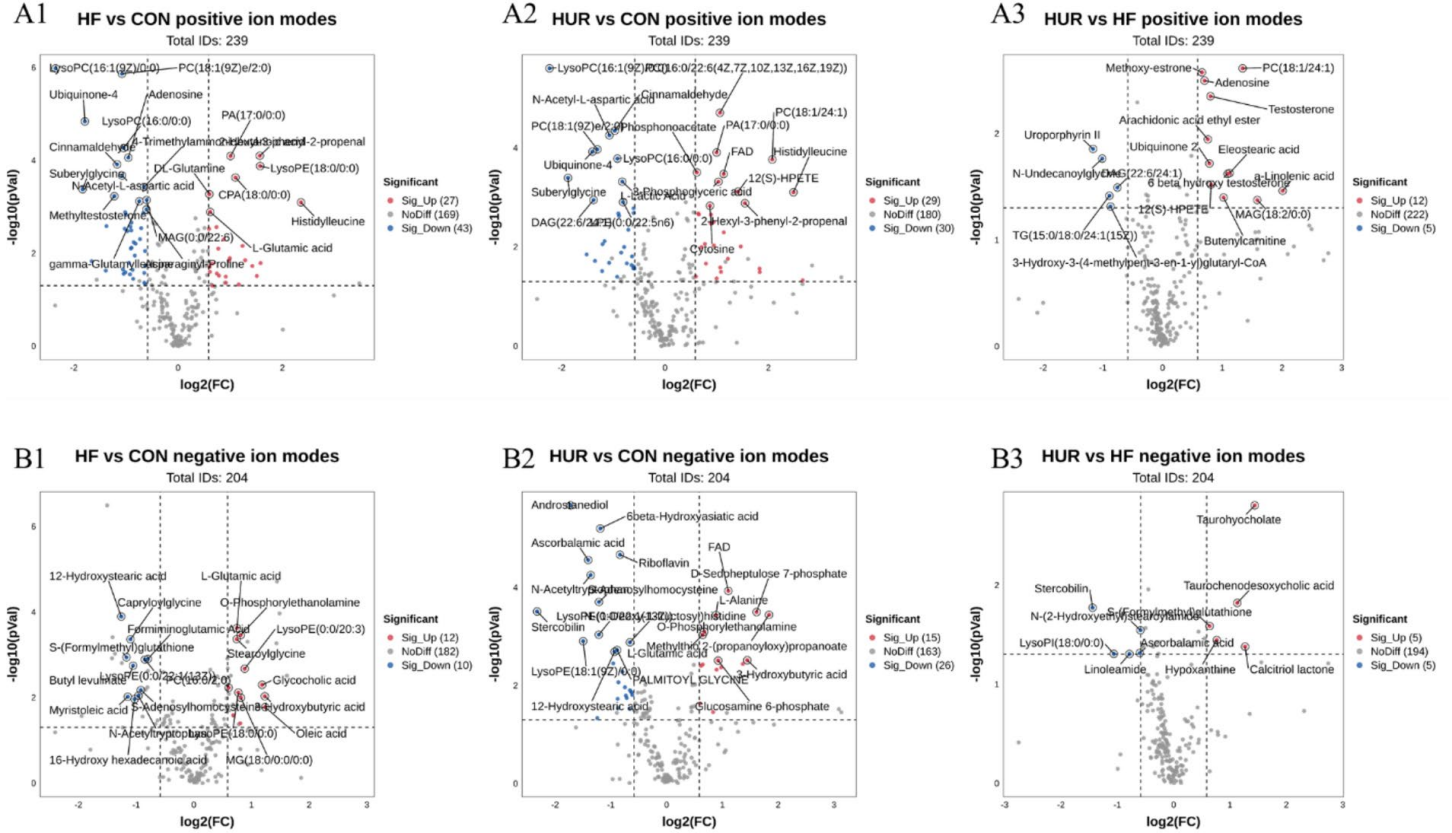
Volcano plot of differential metabolites in the liver between different groups in **(A1–A3)** the positive mode and **(B1–B3)** the negative mode (*n* = 7–8). CON, control diet group; HF, high-fat diet group; HUR, high-fat diet + 0.4 mg/mL uridine in drinking water for the last 4 weeks. A volcano plot indicates the size of the biological effect (fold change) vs. the statistical significance of the result (statistical *p*-value). Each dot represents a metabolite plotted as a function of fold change (log 2 (fold change), x-axis) and statistical significance (−log 10 (*p*-value), y-axis). The pink dots represent selected putative markers with a p-value of ≤0.05 and a fold-change cutoff of > or < 1.5. Remarkable differential metabolites between groups, with red representing significantly upregulated metabolites, blue representing significantly downregulated metabolites, and gray representing insignificant metabolites.

**Figure 8:**
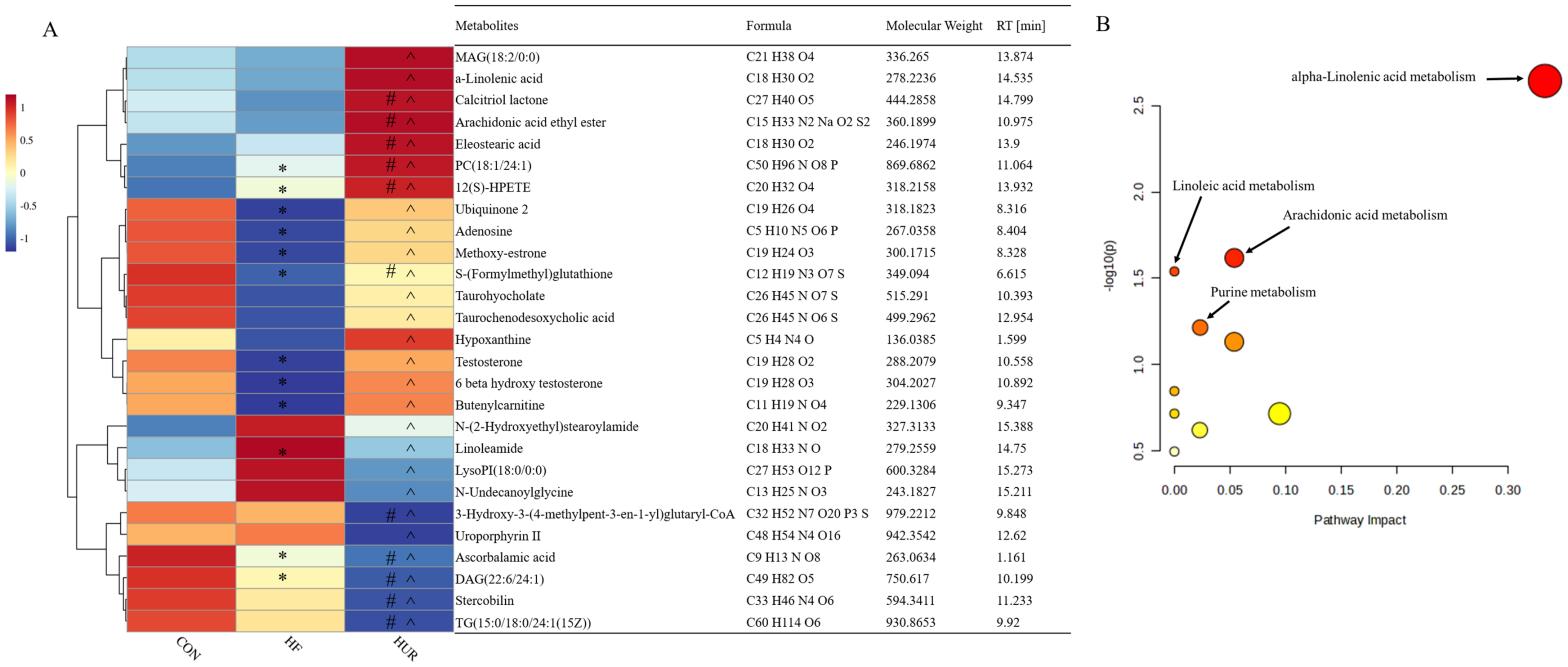
Heatmap of the abundance of liver differential metabolites between the HUR and HF groups **(A)**; overview of metabolic pathway analysis **(B)**. CON, control diet group; HF, high-fat diet group; HUR, high-fat diet + 0.4 mg/mL uridine in drinking water for the last 4 weeks. MAG, monoacylglyceride; PC, phosphatidic acid; LysoPI, lysophosphatidylinositol; DAG, diacylglycerol; TG, triacylglycerol. * HFD vs. CON *p* < 0.05; #HUR vs. CON *p* < 0.05; ^HUR vs. HFD *p* < 0.05.

A total of 443 known metabolites, including 239 in the positive ion mode and 205 in the negative ion mode, were identified in the liver of mice using LC-MS metabolomic analysis. The clustering analyses based on partial least squares discriminant analysis (PLS-DA) were used to discriminate the metabolic profiles among groups (Figure 6). Subsequently, the permutation test verified that the model was not “over-fitting.” The samples in the CON, HF, and HUR groups were separated in both positive and negative ion modes, indicating that the overall metabolic state of the mice changed after treatment. For further analysis, the candidate markers were selected by examining the volcano plot and considering a fold-change (FC) threshold of 1.5, VIP > 1, and *p*-value less than 0.05 (Figure 7). There were 88 differential metabolites between the CON and HF groups, 100 between the CON and HUR groups, and 37 between the HF and HUR groups. To further understand the metabolic pathways in the HUR group mice (Figure 8), metabolic pathway analyses of 37 different enriched metabolites were performed using MetaboAnalyst 5.0.^1^ The HUR group metabolites revealed several metabolism pathways, including *α*-linolenic acid metabolism, linoleic acid metabolism, arachidonic acid metabolism, and purine metabolism, compared with the HF group.

Metabolomics measures metabolic changes in the liver, and fatty acids (α-linoleic acid and arachidonic acid) can reduce hepatic steatosis and improve liver function in HF diet-fed mice (42, 43). In addition, dietary nucleotides affected levels of linoleic and arachidonic acid in rats with liver cirrhosis (44). Similarly, uridine supplements increased the percentage of polyunsaturated fatty acids in the liver (15). In the current study, the levels of α-linolenic acid and 12(S)-HPETE were higher in the HUR group than in the HF group, and uridine restored the decrease of arachidonic acid caused by HF diet feeding. It revealed that unsaturated fatty acids play an important role in the regulation of liver lipid metabolism disorder by uridine. Testosterone is a metabolic hormone that regulates the expression of key targets of lipid and glucose metabolism and may reduce fat deposition in the liver (45). In this study, the HF diet decreased the testosterone level in the liver, and uridine restored it to normal levels. Uridine metabolism affected the levels of adenosine; the deletion of uridine phosphorylase (an enzyme that catalyzes the phosphorolysis of uridine into uracil) increased adenosine to a lesser extent (46). Notably, hypoxanthine (an adenosine metabolite) showed impaired rhythmicity due to the decrease in peak concentration at ZT8 in the HF group (47). The results showed that uridine alleviated the decrease of adenosine and hypoxanthine induced by the HF diet. It may suggest that there is an interactive effect on the metabolism of different kinds of nucleosides or nucleobases. In addition, the levels of chenodeoxyglycocholic acid and taurodeoxycholic acid were altered by nucleotide supplementation in alcohol-treated rats (48). The results from the present study also showed that uridine affected the level of taurochenodeoxycholic acid. Further mechanism research can be conducted using the mouse liver organoid model, such as functional validation of differential metabolites and study of nucleotide metabolism pathways discovered in this experiment.

## 4 Conclusion

In summary, uridine supplementation exerted significant anti-obesity effects in diet-induced obese mice, including reductions in final body weight, intra-abdominal white adipose tissue weight, and serum and liver lipid accumulation. In addition, uridine administration modulated the expression of genes involved in lipid transport and pyrimidine metabolism. Furthermore, metabolomic analysis revealed significant alterations in liver metabolites, particularly those associated with arachidonic acid and *α*-linolenic acid metabolism. These findings collectively demonstrate that uridine ameliorates HF diet-induced obesity by modulating hepatic lipid and nucleoside metabolism.

## Data Availability

The original contributions presented in the study are included in the article/supplementary material, further inquiries can be directed to the corresponding author.

## Footnotes

^1^ https://www.metaboanalyst.ca/
